# ASK2 Bioactive Compound Inhibits MDR *Klebsiella pneumoniae* by Antibiofilm Activity, Modulating Macrophage Cytokines and Opsonophagocytosis

**DOI:** 10.3389/fcimb.2017.00346

**Published:** 2017-08-04

**Authors:** Cheepurupalli Lalitha, Thiagarajan Raman, Sudarshan S. Rathore, Manikandan Ramar, Arumugam Munusamy, Jayapradha Ramakrishnan

**Affiliations:** ^1^Centre for Research in Infectious Diseases, School of Chemical and Biotechnology, SASTRA University Thanjavur, India; ^2^Department of Zoology, University of Madras Chennai, India

**Keywords:** antibiofilm, immunomodulation, MDR *K. pneumoniae*, biofilm, cytokines

## Abstract

The emergence and spread of pathogens harboring extended spectrum beta-lactamase (ESBL) like carbapenem resistant Gram negative bacteria are the major emerging threat to public health. Of particular concern *Klebsiella pneumoniae* carbapenamase- producing strains have been recorded worldwide. Catheter associated urinary tract infections (CAUTI) caused by *K. pneumoniae* are significantly associated with morbidity and mortality. Hence the present work was aimed to develop a strategy for addressing these issues through an innovative approach of antibiofilm and immunomodulation. These two independent activities were analyzed in a *Streptomyces* derived ASK2 bioactive compound. While analysing the effect of sub-minimum inhibitory concentrations (sub-MICs), 0.5x of Minimum Inhibitory Concentration (MIC) was found to be more effective in preventing biofilm formation on coverslip and silicone catheter. The minimum biofilm eradication concentration (MBEC) was found to be 15-fold higher MIC with eradication of 75% of 3 day old biofilm. Apart from its antibiofilm potential, ASK2 also acts as an opsonin and enhances phagocytic response of macrophages against multidrug resistant *K. pneumoniae*. In addition, ASK2 resulted in elevated levels of nitric oxide generation by the macrophages and has a stimulating effect on IL-12, IFN-γ, and TNF-α proinflammatory cytokines. The opsonic role of ASK2 and its potential in modulating proinflammatory cytokines secreted by macrophages implies the importance of ASK2 in modulating cellular immune response of macrophages against MDR *K. pneumoniae*. The present study proposes ASK2 as a promising candidate for treating MDR *K. pneumoniae* infections with its dual properties of antibiofilm and immunomodulatory activities.

## Introduction

Carbapenem resistant enterobacteriaceae (CRE) such as *K. pneumoniae* and *E. coli* are opportunistic nosocomial bacteria that can cause different health care associated infections such as urinary tract infections (UTI), intra abdominal infections, wound infections and meningitis (Paterson, 2000; Kumari et al., 2011). Their ability to adhere and grow as biofilm on materials such as catheter is crucial for progression of infection. Urinary catheters are routinely used medical devices and are highly prone to colonization by UTI bacteria. Nearly 60–80% of patients undergoing long term catheterization are susceptible to the development of catheter associated urinary tract infection (Lo et al., 2014). The most common UTI microbes are *E. coli, K. pneumoniae, Pseudomonas* sp., *Candida* sp., *Enterococcus* sp. etc. (Brennan et al., 2014; Nicolle, 2014). Besides, *K. pneumoniae* is the major cause of infections in catheterized patients, and hence categorized as one of the top eight significant nosocomial pathogens. *K. pneumoniae* is an encapsulated bacteria that colonizes the human gastrointestinal tract, skin, nasopharynx and urinary tract. It is characterized by the presence of major virulence factors such as capsule (Siu et al., 2012), type 1 and type 3 pili, KPF-28 fimbriae (Di Martino et al., 2003), lipopolysaccharide (LPS) (Vuotto et al., 2014) and siderophores (Schembri et al., 2005). Among these, capsular polysaccharide is a significant virulence factor in *K. pneumoniae* that enable host defense evasion mechanism (Yu et al., 2008). The capsule protects the bacterium from phagocytosis (Troy, 1992; Ko et al., 2013). In addition, the capsule material that cover the underlying LPS prevent the activation of complement proteins to form membrane attack complex and cell lysis (Álvarez et al., 2000). Also, capsular polysaccharide is one of the important bacterial components that help them to form biofilm on solid surfaces such as indwelling catheters (Murugan et al., 2016). The deposition of urinary components on catheters favors the microbes to colonize, divide, followed by production of extracellular matrix, interspecies communication and dispersion of cells from biofilm to complete the cycle. The dispersed cell exists in planktonic state and the biofilm cycle continued (Prasad et al., 2009). Microbes in biofilm state have certain survival advantages such as resisting immune defenses and antimicrobial actions (Fàbrega et al., 2014). Collectively, biofilm formation, antiphagocytic mechanism, production of ESBL, and carbapenamase are the major cause of concern that makes clinicians to be highly dependent on polymyxins and tigecycline for treatment of these infections, in spite of their high toxicity (Arnold et al., 2007). Hence several attempts have been made to prevent biofilm formation by instilling catheters with higher concentrations of antimicrobial agents. However, the use of antimicrobial lock therapy is potentially toxic to the patient due to diffusion of the lock solution into the systemic circulation, which is in addition to the development of antimicrobial resistance (Justo and Bookstaver, 2014).

Alternatively, some new beta-lactamase inhibitors in combination with antimicrobial agent have been developed for the treatment of complicated UTI infections. However, the emergence of new drug resistant phenotypes are expected to be more prevalent in the future (Ventola, 2015). Also, most such strategies are replete with adverse side effects and may not be favorable though the benefits far outweigh the risks. Nevertheless, one way forward to tackle such complicated UTI infections with no or minimal side effect is immunotherapy. Majority of the immunotherapy strategies rely on immunomodulation of the host's immune system to prime it against the pathogen. This method has many advantages including lack of side effects and resistance development. More importantly such treatment to some extent can be catered to suit an individual patient's need. Hence the present work was aimed to develop a strategy to address these issues through an innovative approach of antibiofilm and immunomodulation for treating biofilm based infections of Multi Drug Resistant (MDR) *K. pneumoniae*.

In our previous study we described the isolation and partial characterization of bioactive compound ASK2 from *Streptomyces* sp. ASK2 having potential antagonistic activity against MDR *K. pneumoniae*. The highly polar ASK2 compound was found to be an aromatic compound with aliphatic side chain molecule having a molecular weight 444.43 Da. FTIR showed the presence of OH and C=O as functional groups (Cheepurupalli et al., 2017). In this report, we extended the investigation to evaluate antibiofilm and immunomodulatory properties of ASK2. First, we studied the antibiofilm effect of ASK2 bioactive compound in preventing and eradicating *K. pneumoniae* biofilm formed on coverslip and urinary catheter. In addition, the potential of ASK2 to stimulate nitric oxide production, phagocytosis and modulation of macrophage cytokine gene expression during phagocytosis were studied. To the best of our knowledge this is the first report to show a dual antibiofilm and immunomodulatory potential of a bioactive compound against MDR *K. pneumoniae*.

## Materials and methods

### Strains and storage

Two different strains of MDR *K. pneumoniae* were used in this study. The sensitivity pattern of clinical MDR *K. pneumoniae* was described in our previous study. The reference strain, NDM *K. pneumoniae* 05-506 was purchased from Microbial Culture Collection Centre at Pune, India (Yong et al., 2009).

### ASK2 compound

In our previous study a potential *Streptomyces* sp. ASK2 was isolated from rhizosphere soil of a medicinal plant. Upon multistep HPLC purification the active compound exhibiting antagonistic activity against MDR *K. pneumoniae* was identified and compared with oxytetracycline (OTC) (produced by *S. rimosus*, the closest representative species of ASK2 strain). The HPLC comparison, IR spectrum, ESI-MS and ^1^H NMR data of the purified compound was different from that of OTC. Hence the bioactive compound isolated from *Streptomyces* sp.ASK2 is warrant to be a new compound (Cheepurupalli et al., 2017).

### Biofilm formation on coverslip and silicone catheter

*K. pneumoniae* strains were separately grown overnight in nutrient broth at 37°C. The cells were then collected by centrifugation at 5,000 × g for 10 min, washed with PBS and resuspended in 10 ml of nutrient broth. Coverslip of 22 mm × 22 mm were placed in sterile 6 well polystyrene tissue culture plates and were inoculated with 500 μl (0.08 OD) of bacterial suspension, and incubated at 37°C for 72 h. The non-adherent cells were removed and biofilm were quantified by Crystal Violet assay (CV), MTT assay (3-(4,5-Dimethylthiazol-2-Yl)-2,5-Diphenyltetrazolium Bromide) and CFU (Colony Forming Unit) estimation.

Catheter discs of 2 × 2 cm^2^ which were cut from silicone foley urinary catheter and surface sterilized with 75% of isopropanol. The sterile catheter discs were then immersed in artificial urine medium for 30 min (Burton et al., 2006). After pre-treatment, the discs were transferred to 6 well culture plate and biofilm were formed and quantified as described below (Pérez et al., 2010; Doll et al., 2016). All experiments were performed in duplicates or triplicates on two independent days.

#### Biofilm quantification methods

##### Crystal violet assay

Biofilm was quantified according to the method of Kaur et al. (2016). Briefly biofilms were fixed with methanol and stained with 0.1% of CV for 15 min, followed by rinsing with sterile distilled water and subsequent destaining with 33% of glacial acetic acid. The absorbance of the biofilm was measured at 590 nm by using spectrophotometer.

##### CFU determination

To confirm the antibiofilm effect of ASK2, the surviving bacterial populations in the biofilm was estimated using the viable plate count method. After 24 h treatment by ASK2, the coverslip and discs were washed with sterile PBS, and the adherent biofilm was scraped and serially diluted using PBS and plated on UTI agar plates and incubated at 37°C for 48 h.

##### MTT cell viability assay

After treatment of biofilm with ASK2, the medium from the wells were removed carefully and MTT assay was performed. Briefly, MTT solution was added along with media and the plates were incubated for 4 h at 28°C. At the end of the incubation, 200 μl of DMSO was added and allowed to react for 45 s and then supernatant transferred into 96 well plate. The readings were obtained at 540 nm using microplate reader.

### Prevention of biofilm formation on coverslip and catheter by ASK2

The efficacy of sub-MICs of ASK2 on biofilm prevention was determined. The above protocol was followed for biofilm formation simultaneously the cells were challenged with sub-MICs of ASK2 (0.5x, 0.25x, 0.125x MICs). The plates were incubated at 37°C and the biofilm prevention was recorded at 24 h. OTC (CMS653, Himedia) was used as drug control to determine comparative antibiofilm activity.

### Determination of minimum biofilm eradication concentration (MBEC)

Biofilms were formed on coverslip and catheter disc as described above. The 3 day old biofilm was treated with 2.5x, 5x, 10x, 15x MICs of ASK2 and OTC for 24 h at 37°C. After treatment, the biofilm was washed with PBS and biofilm was quantified and relative biofilm inhibition was expressed in mean percentage.

### Microscopic assessment

For microscopic assessment, biofilms were treated with biofilm eradication concentration (15x MIC) and biofilm prevention concentration (0.5x MIC). After treatment, the biofilms were washed with PBS stained with FITC-Con A and propidium iodide and observed in CLSM.

To determine the morphological changes with respect to capsule thickness in ASK2 treated biofilm, a similar staining protocol was followed and viewed in a fluorescent microscope. Capsule thickness was recorded by measuring diameter of whole cell, including capsule and cell limited by cell wall by using Image J1.4.3.67v software (Rathore et al., 2016).

### Cytotoxicity assay of ASK2

The *in vitro* cytotoxicity assays were performed in macrophages (J774.A.1 and Raw264.7) and normal human epithelial embryonic kidney cell lines (HEK-293), procured from National Center for Cell Science, Pune, India. ASK2 concentrations of 8, 16, 32 μg/ml were analyzed for its cytotoxic effect on macrophage cell lines by performing MTT assay at 12 h time intervals for 24 h. Similarly in HEK- 293 cell line, ASK2 concentrations (1 μg/ml to 300 μg/ml) were used.

### Phagocytosis assay

Macrophages (J774.A.1 and RAW 264.7) were plated at a density of 10^5^ cell/ml with complete media without antibiotics on acid washed coverslip (22 × 22 mm^2^) placed in 6 well plate. The details of different treatment groups are provided in Supplementary File 1. The phagocytosis assay was compared between non-activated and macrophages activated with 3 μg/ml LPS and 100 pmol IFN-γ (2 h) or ASK2. The macrophages were then presented with heat killed (56°C for 1h) clinical *K. pneumoniae* (0.05 OD) that were either non-opsonized or opsonized (8 μg/ml or 16 μg/ml with ASK2 for 4 h). The bacterial cells were then washed with 1XPBS, and were resuspended in DMEM medium without serum and antibiotics. Phagocytosis was performed with 1:10 ratio of macrophage:bacteria and phagocytosis rate was analyzed at different time intervals. The cells were then fixed with 1.25% glutaraldehyde for 5 min and observed for internalized bacterial cells.

$$\begin{array}{l} \text{Phagocytosis rate }(\%)\\ \;=[(\text{Number of macrophages with internalized bacteria}/\\ \;\text{Total number of macrophages})\;\times \;100\;] \end{array}$$

### Nitric oxide assay

Macrophage cells were serum starved for 6 h followed by the incubation at a concentration of 1 × 10^4^ cells/ml in sterile 96 well plate with ASK2 at concentrations of 8, 16, and 32 μg/ml or LPS (1, 2, and 3 μg/ml) with IFN-γ (100 picomol) for 24 h. After incubation, the supernatant was used for nitric oxide assay using Griess reagent and measured at 540 nm. The quantity of nitrite was determined using sodium nitrite in a standard curve (Amano and Noda, 1995).

### Cytokine gene expression analysis

The role of ASK2 in modulating cytokine gene expression was ascertained using J774.A.1 and RAW264.7 macrophages. For transcription pattern analysis, phagocytosed cells were washed with serum free medium and total RNA was extracted using TRIzol method (30006, Takara) and 2–10 μg of RNA sample were converted into cDNA by one step RT-PCR Kit (210201, Qiagen). Cytokine genes amplifications from templates (1:20) with target primers (2:20), and (10:20) Emerald Amp GT PCR master mix (RR310, Clontech, Takara). The protocol for PCR were as follow: initial denaturation at 95°C for 5 min, followed by cycles of 95°C for 30 s, annealing at 61°C (GAPDH and IL-12), 57°C (IL-4), 58°C (TNF-α), 55°C (IFN-γ) for 30 s and extension at 72°C for 30 s and final extension at 72°C for 7 min. Primer sequence for PCR is listed in Table 1. Semi-quantitative gene expression was performed. Expressed PCR product was quantified by measuring the intensity peak area of amplified DNA band using ImageJ software (Rathore et al., 2016; Supplementary File 2).

**Table 1:** Primer sequence.

**Primer**	**Type**	**Sequence**	**Product size**	**Cycle**	**References**
IL-4	Forward	5′-TCGGCATTTTGAACGAGGTC-3′	216	30	Sisto et al., 2003
IL-4	Backward	5′-GAAAAGCCCGAAAGAGTCTC-3′			
IL-12	Forward	5′-CGTGCTCATGGCTGGTGCAAAG-3′	220	30	Sisto et al., 2003
IL-12	Backward	5′-CTTCATCTGCAAGTTCTTGGGC-3′			
IFN-γ	Forward	5′-GCTCTGAGACAATGAACGCT-3′	227	35	Munder et al., 1998
IFN-γ	Backward	5′-AAAGAGATAATCTGGCTCTGC-3′			
TNF-α	Forward	5-ATGAGCACAGAAAGCATGATC-3′	276	30	Sisto et al., 2003
TNF-α	Backward	5′-TACAGGCTTGTCACTCGAATT-3′			
GAPDH	Forward	5′-ACAGTCCATGCCATCACTGCC-3′	266	45	Stephens et al., 2011
GAPDH	Backward	5′-GCCTGCTTCACCACCTTCTTG-3′			

### Statistical analysis

All experiments were performed in duplicates or triplicates, and data analysis were executed in Graphpad prism 6.0 software. One way ANOVA followed by multiple comparison tests were performed and significant difference between the samples were calculated by unpaired parametric student *t*-test (*p* < 0.05). All graphs were prepared with Graphpad prism 6.0 and were expressed as the mean ± standard deviation (SD) of triplicates Significant difference between the samples was calculated by unpaired parametric student *t*-test (*p* < 0.05).

## Results

### MDR *K. pneumoniae* strains are proficient biofilm producers

**Figure 1:**
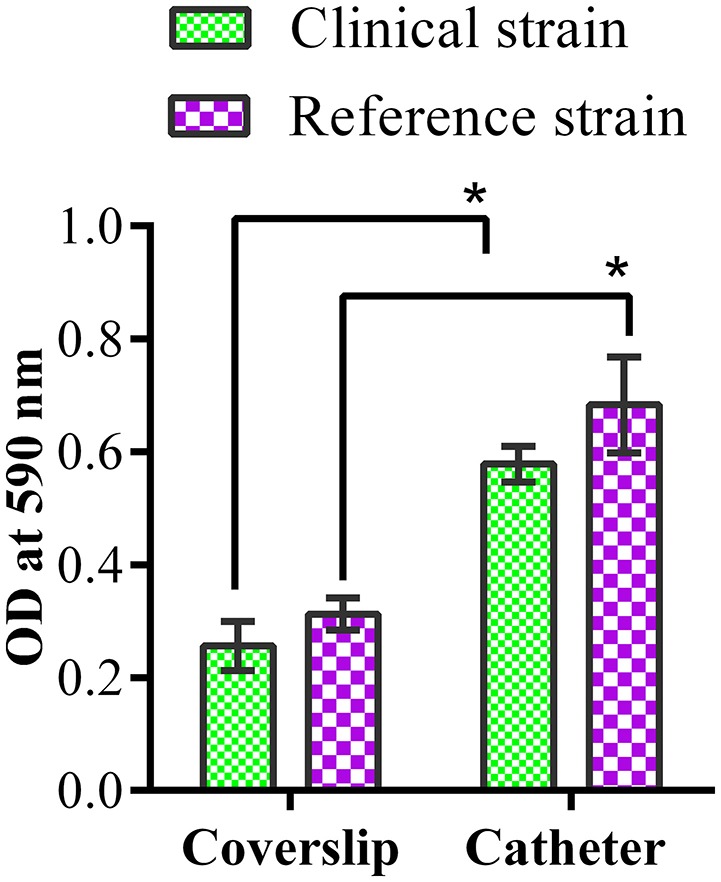
Biofilm quantification by crystal violet assay. Quantification of clinical and reference *K. pneumoniae* biofilm formation on coverslip and catheter. ^*^ represents the significant difference (*p* < 0.05).

CV assay revealed stronger biofilm formation by both the strains on catheter than the coverslip (Figure 1). Biofilm formation was higher at 72 h of incubation for both the strains.

Thus, the clinical and reference strains of *K. pneumoniae* used in this study were characterized as efficient biofilm producers on catheter and coverslip which make them suitable for screening antibiofilm effects of ASK2 compound.

### Biofilm inhibition by sub-MICs of ASK2

**Figure 2:**
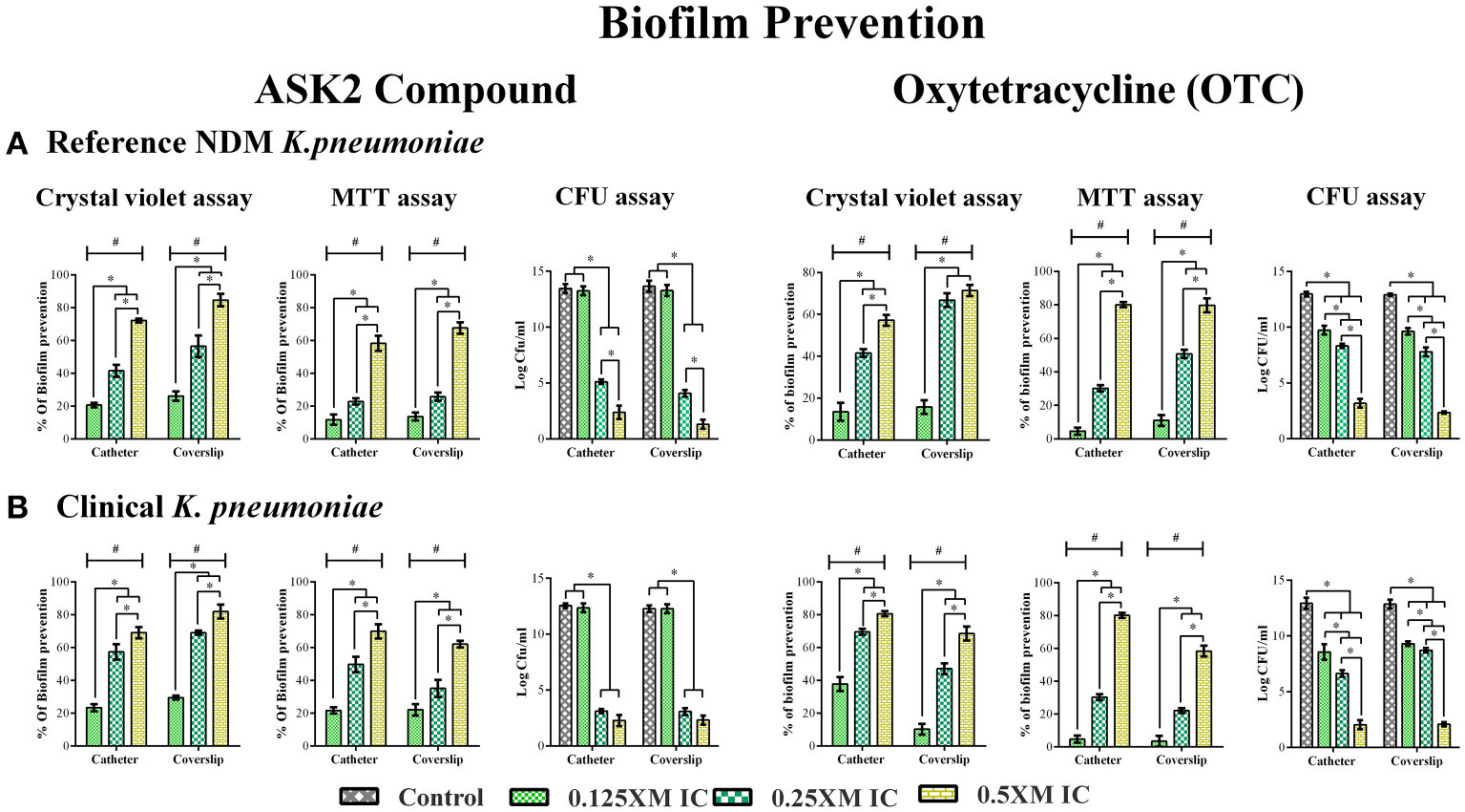
Effect of Sub- MICs of ASK2 in biofilm prevention of *K. pneumoniae*. *K. pneumoniae*, reference strain **(A)** and clinical strain **(B)** and were grown in coverslip and silicone catheter in the presence of different sub- MICs (0.125, 0.25, 0.5x MICs) of ASK2 and OTC. After treatment, biofilm were quantified by CV assay (A_590_), MTT assay (A_540_), and CFU assay (UTI agar medium). Duplicate samples were used for each treatment, and the experiment was repeated three times. Student *t*-test was performed and data were represented as mean ± SD. ^*^ represents the significant difference between the concentrations and # relative to control (*p* < 0.05).

Biofilm inhibitory property of ASK2 was evaluated by treating clinical and reference strains with different sub-MICs of ASK2 at 37°C for 24 h. Apparently from CV assay, 0.5x MIC (8 μg/ml) significantly reduced biofilm formation of clinical strain by 70% (catheter) and 80% (coverslip). Similar kind of observation was recorded for reference strain, with maximum reduction of 9.81 log cfu/ml of both the strains. Similarly, MTT assay showed biofilm prevention of clinical strain by 70% (catheter) and 62% (coverslip). In case of reference strain 60% (catheter) and 67% (coverslip) were observed, with maximum reduction of 9 log cfu of both the strains. Collectively CV, MTT assay and cfu counting suggested that sub-MICs prevent biofilm formation in a dose dependent manner and 0.5x MIC was found to be more effective for both the strains (Figure 2).

### Determination of minimum biofilm eradication concentration (MBEC)

**Figure 3:**
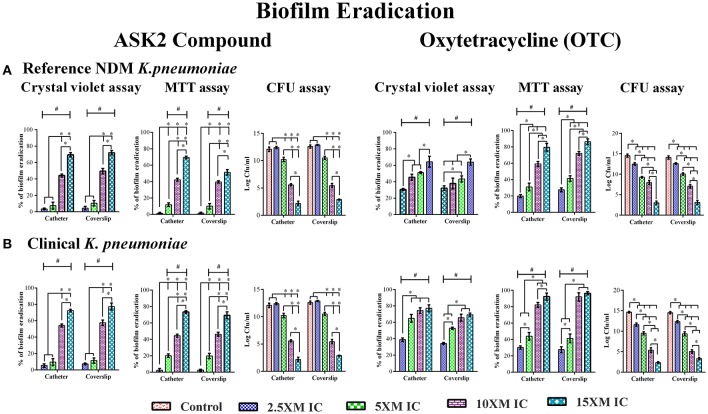
MBEC determination. Biofilm eradication of reference strain **(A)** and clinical strain **(B)**. The 3 day old preformed biofilm on coverslip and catheter were treated with different concentrations (2.5x, 5x, 10x, and 15x MICs) of ASK2 and OTC. After 24 h, biofilm cells were quantified by CVassay (A_590_), MTT assay (A_540_), and CFU assay (UTI agar medium). Duplicate samples were used for each treatment, and the experiment was repeated three times. Student *t*-test was performed and data were represented as mean ± SD. ^*^ represents the significant difference between the concentrations and # relative to control (*p* < 0.05).

When preformed biofilm were treated with higher concentrations of ASK2 (2.5x to 15x MIC), the CV assay showed biofilm eradication of clinical strain by 79% (coverslip) and 75% (catheter) at 15x ASK2 (240 μg/ml). Similarly, 70% of biofilm eradication of reference strain was observed on both the solid supports. In addition, MTT assay also confirmed biofilm eradication of both the strains by maximum of 75% at 15x ASK2. The viability counting showed the reduction of 9.9 log cfu/ml on UTI agar medium. The MBEC of ASK2 of both the strains is 240 μg/ml, that is 15-fold higher than MIC of ASK2 was required to eradicate the maximum of 75% *K. pneumoniae* biofilm. Figure 3 showed that ASK2 has similar antibiofilm effect like that of OTC.

### Confocal and fluorescent microscopy

**Figure 4:**
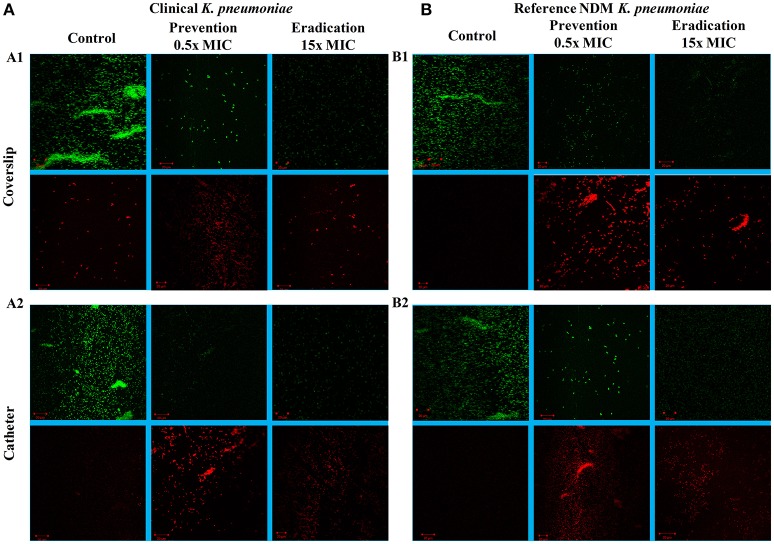
CLSM images. Antibiofilm activity of ASK2 against clinical **(A)** and reference **(B)** *K. pneumoniae*. For biofilm prevention, ASK2 (0.5x MIC) was added to the medium from the beginning of the experiment. For biofilm eradication, ASK2 (15x MIC) was added to 3 days old biofilm formed on coverslip **(A1)** and catheter **(A2)**. After 24 h, biofilm were stained with Con-A conjugated FITC (30 μg/ml) and propidium iodide (1 μg/ml) prior to confocal imaging. Untreated sample served as control. Green fluorescence indicates live microbes and red fluorescence shows dead cells. Presence of less dense cells are visualized in treated samples when compared with control.

**Figure 5:**
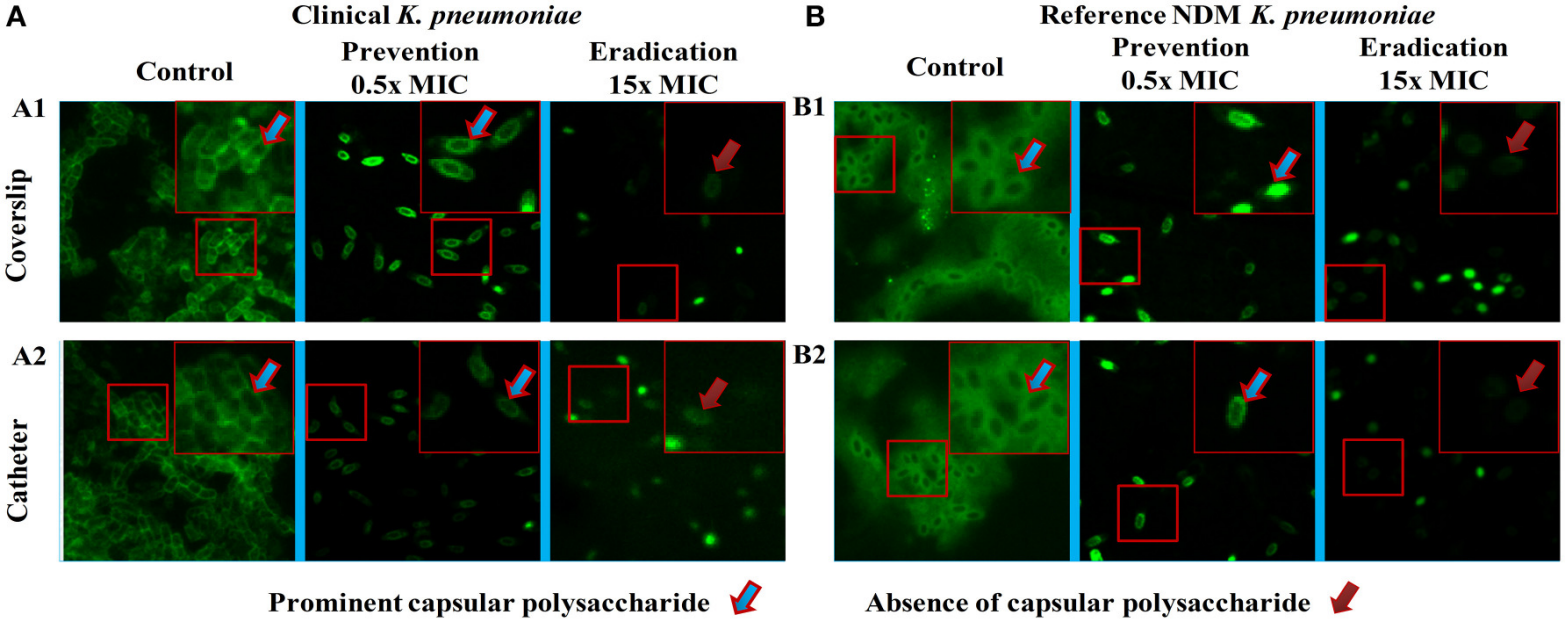
Fluorescent microscopy. Morphology analysis using fluorescent microscopy. Capsule thickness was measured by ImageJ 1.4.3.67v software. Capsule thickness = [(capsule size of control − treated)/control] × 100. Ten cells were measured for each determination and average was calculated. Disrupted morphology with reduced capsule size and cell size were observed in treated samples. Approximately 50% significant capsule reductions were observed for both **(A)** clinical and **(B)** reference strains. Capsule size reduction = {[(Capsule size of control − treated)/control] × 100}.

The images of the positive control (Figure 4) showed the presence of densed biofilm (green cells) on coverslip and catheter disc, whereas the treated samples were less densed (red cells), inferring the antibiofilm activity of ASK2. The reduction of capsule size surrounding the bacterial cell are clearly visualized in treated sample when compared with positive control (Figure 5). The capsule size of both strains were reduced to 50% and notable reductions in cell numbers and cell size were also recorded (Supplementary File 3).

### *In vitro* toxicity studies

**Figure 6:**
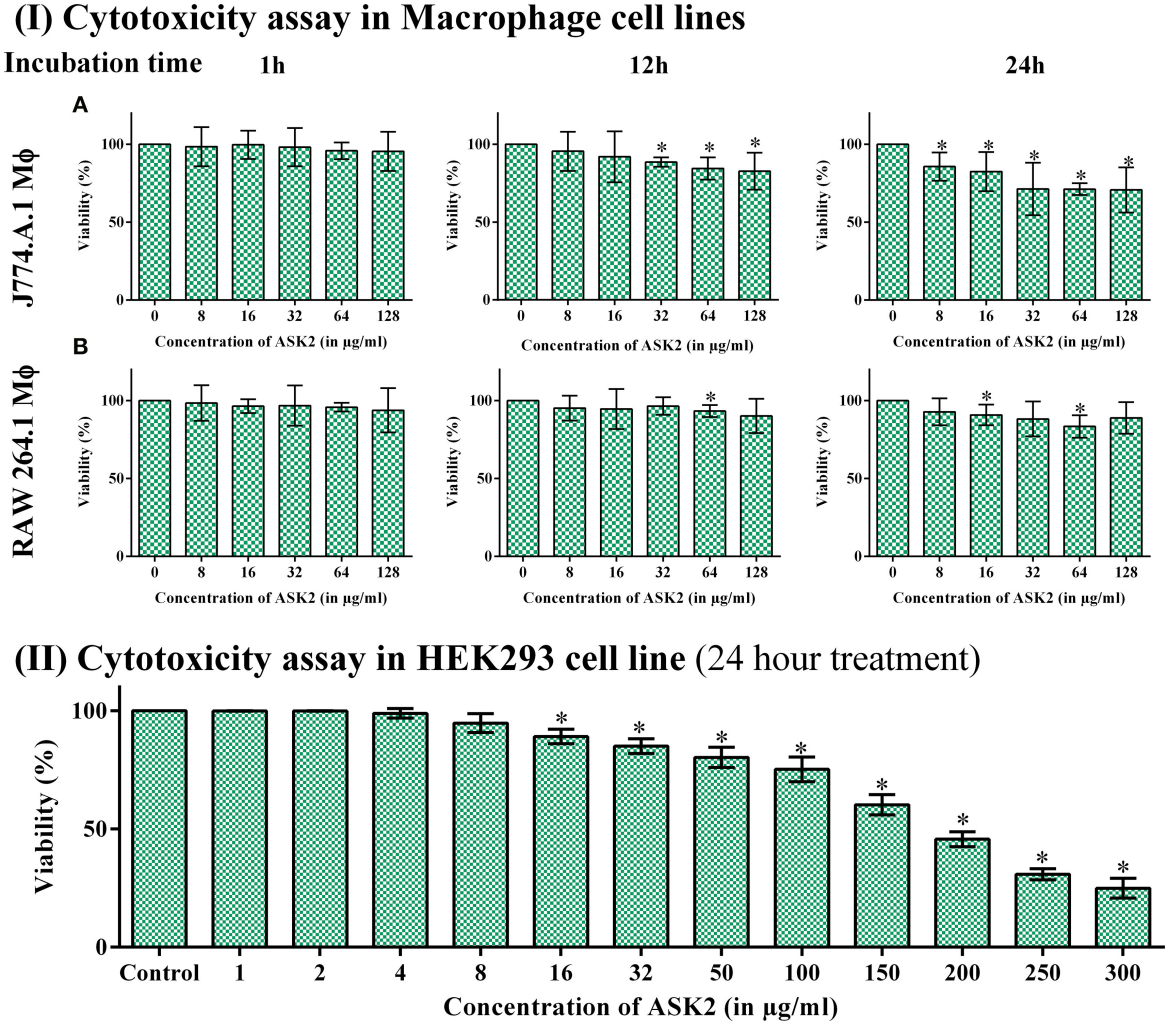
**(I)** Cytotoxicity assay in macrophage cell line. Cytotoxicity of ASK2 at different concentrations and time intervals in J774.A.1 Macrophage cell line **(A)** and RAW 264.7 Macrophage cell line **(B)**. No toxicity was observed in RAW 264.7 up to 24 h incubation with 88% viability and 71% viability for J774.A.1. No significant difference was observed at different incubation time and concentrations. **(II)** Cytotoxicity assay in HEK 293 cell line: No cytotoxic effects upto 100 μg/ ml in HEK 293 cell line, cell toxicity was induced in a concentration dependent manner. Student *t*-test was performed. ^*^ represents the significant difference (*p* < 0.05).

While analyzing the cytotoxic effect of ASK2 on RAW264.7 and J774.A.1 cell lines, cell viability of 88 and 71% were retained, respectively, even at highest concentration of ASK2 (128 μg/ml) at 24 h (Figure 6I).

Similarly, with HEK-293 cell lines, there was no cytotoxic effects upto 100 μg/ml, and toxic effects were observed at ≥150 μg/ml (9x MIC). Figure 6II infers that cell toxicity was induced in a concentration dependent manner in HEK-293 cell line.

### ASK2 acts as opsonin and enhances phagocytosis

**Figure 7:**
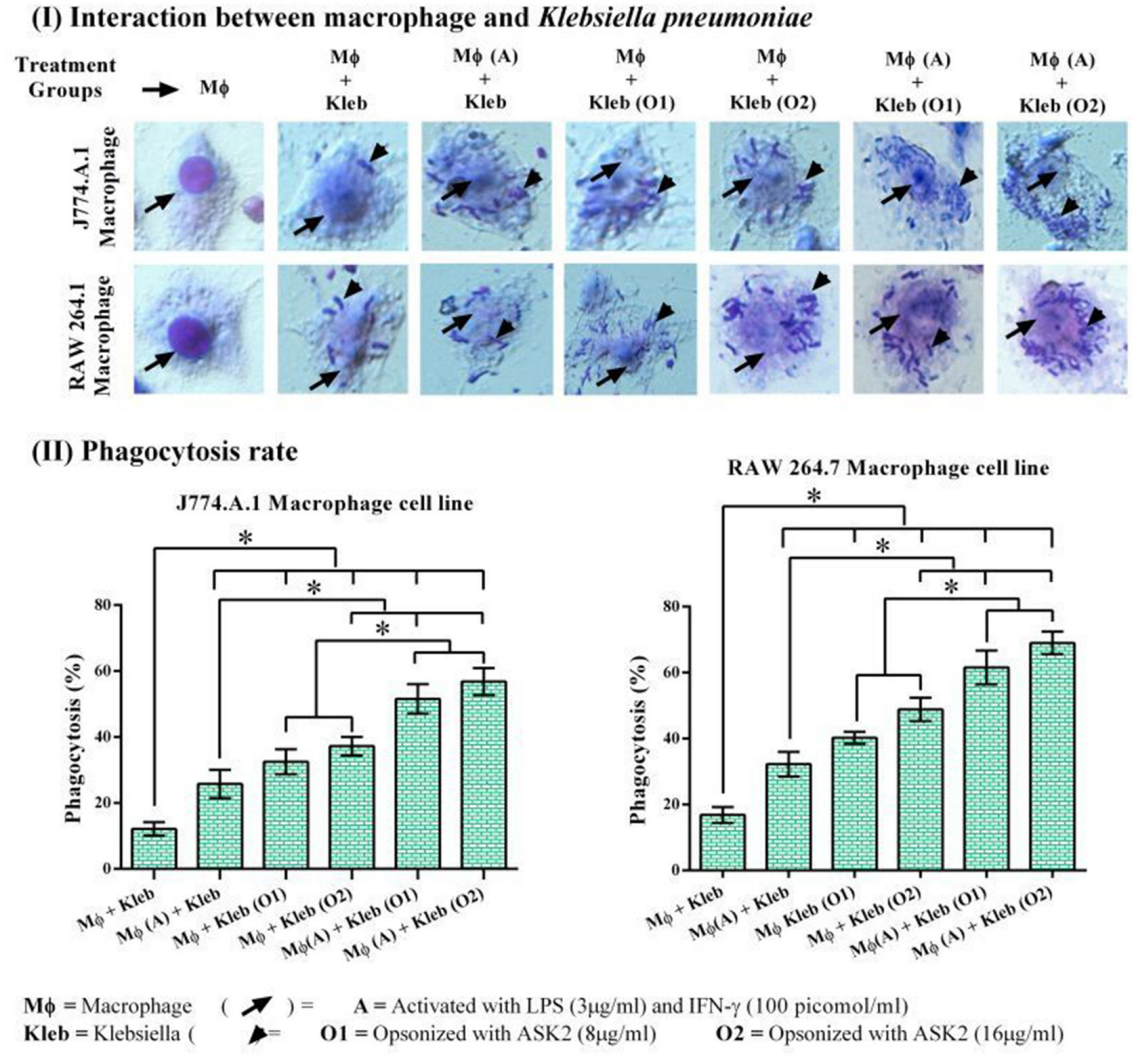
**(I)** Host pathogen interaction and phagocytosis assay. Interactions of J774.A.1 and RAW 264.7 macrophage cell lines either activated with LPS and IFN-γ or ASK2 with *K. pneumoniae* (clinical strain) non-opsonized or ASK2 (8 and16 μg/ml) opsonized. **(II)** Phagocytosis rate. Increased phagocytosis rate were observed in J774.A.1 (52%) and RAW 264.7 (62%) macrophage cell line. RAW 264.7 showed better phagocytic rate in all treatment groups compared to J774.A.1. The experiment was repeated three times. One way ANOVA followed by multiple comparison tests were performed ^*^ represents significant difference between the treatment groups (*p* < 0.05).

In both RAW264.7 and J774.A.1, activation of macrophages by LPS+IFN-γ resulted in significantly higher phagocytic response against non-opsonized *K. pneumoniae* with 32 and 26% phagocytic activity, respectively. Whereas, non-activated macrophages showed activity of 17 and 12% for RAW 264.7 and J774.A.1, respectively. This suggested that macrophage activation is important for increased phagocytic activity (Vergadi et al., 2017). When target cells were opsonized with 8 μg/ml of ASK2 and presented to non-activated macrophages, RAW264.7 cells showed 40% and J774.A.1 cells showed 32% phagocytic activity. On the other hand when macrophages were first activated by LPS and then presented with opsonized (8 μg/ml) targets, the phagocytic activity significantly increased to 62 and 52% for RAW264.7 and J774.A.1 macrophages, respectively. These results clearly show the effect of opsonization by ASK2 in enhancing phagocytic rate of both macrophages. Similar to this, when concentration of ASK2 was increased to 16 μg/ml there was a corresponding increase in phagocytic response by the activated macrophages. Interestingly the opsonization effect of ASK2 was well evident when non-activated macrophages were exposed to *K. pneumoniae* opsonized with 16 μg/ml of ASK2. This suggests that though macrophage activation by LPS and IFN-γ is important for enhanced phagocytic response, opsonization by ASK2 alone is sufficient to significantly increase phagocytic activity of non-activated macrophages, implying the potential of the compound in modulating cellular immune response of macrophages. Between the two macrophages, RAW 264.7 showed a much better phagocytic rate in all treatment groups compared to J774.A.1 (Figure 7). The relevant statistical analysis data for phagocytosis assays are provided in Supplementary File 4.

### ASK2 stimulates nitric oxide generation in macrophages

**Figure 8:**
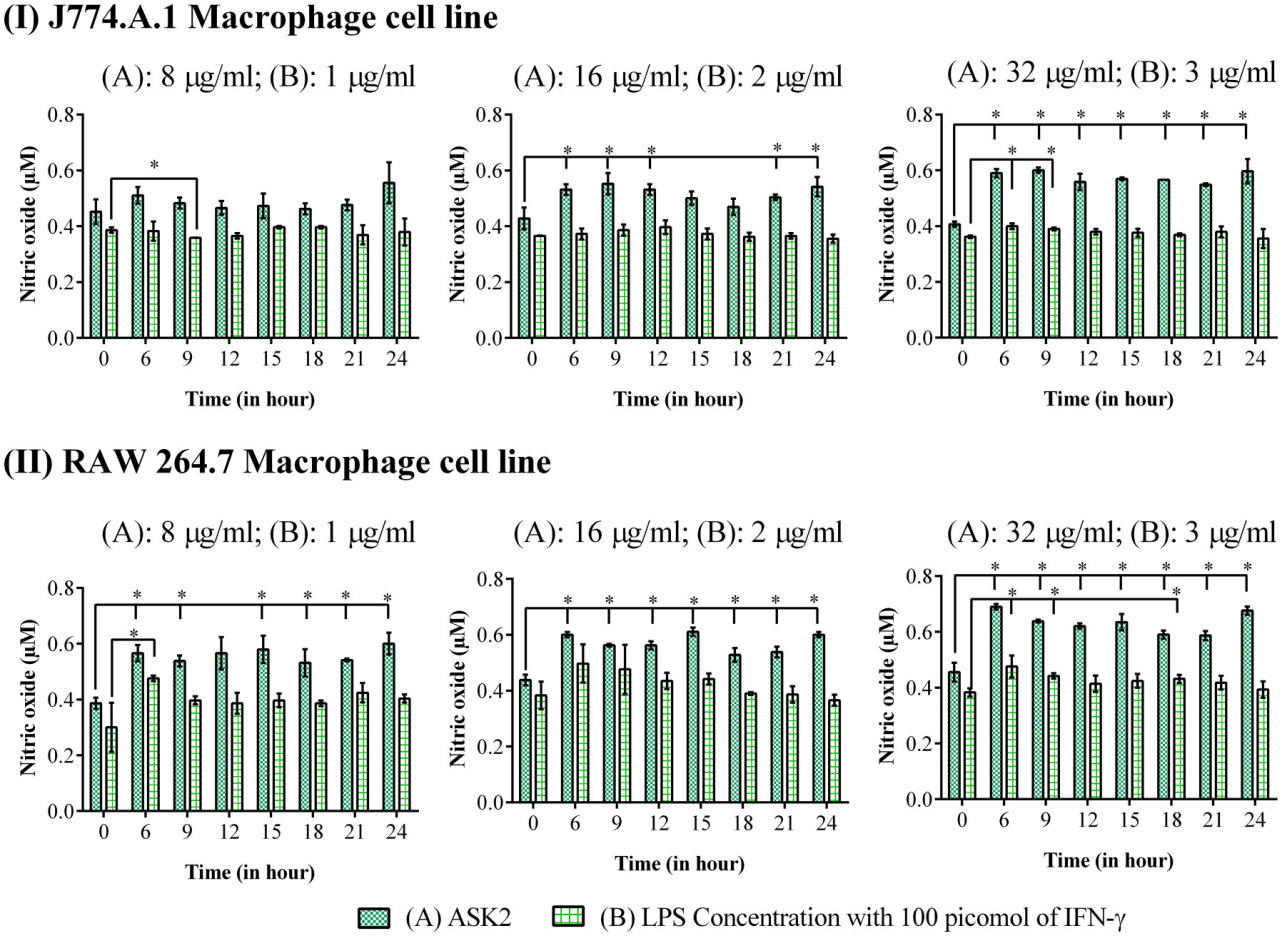
Nitric oxide assay: Elevated levels of nitric oxide production were observed in macrophage cell lines, **(I)** J774.A.1 and **(II)** RAW 264.7 treated at different time intervals with 8, 16 and 32 μg/ml of ASK2 (A) which are comparable with LPS (B) (1, 2, and 3 μg/ml) stimulated macrophages. At 24 h, ASK2 triggered nitric oxide production in both cell lines up to ~0.541 μM. Duplicate samples were used for each treatment, and the experiment was repeated three times. Student *t*-test was performed. ^*^ represent significant difference in nitric oxide production compared to control (*p* < 0.05).

It is clear from Figure 8, that for both macrophage cells, ASK2 was a potent stimulator of nitric oxide generation with significantly (*p* < 0.05) elevated levels even after 24 h of stimulation. This suggests, the role of ASK2 in triggering nitric oxide production by macrophages which could contribute to microbicidal mechanism during phagocytosis.

### ASK2 modulates macrophage cytokine gene expression

Since one of our primary objectives was to look at the immunomodulatory potential of ASK2 compound, we assessed the changes in cytokine gene expression in macrophages during phagocytosis. When J774.A.1 were activated with LPS and IFN-γ, there was significant increase in IL-12 gene expression (0.16-fold) when compared to control J774.A.1 (0.07-fold). However, when J774.A.1 were first treated with ASK2 and then exposed to *K. pneumoniae*, there was an increase in IL-12 gene expression (0.65-fold) and a similar level was also seen with J774.A.1 that were exposed to *K. pneumoniae* opsonized with ASK2 (0.48-fold). A similar response was also observed with RAW 264.7 wherein, RAW 264.7 exposed to LPS (0.23-fold), *K. pneumoniae* (0.17-fold) or ASK2 (0.14-fold) alone showed a slight increase in IL-12 production. On the other hand RAW 264.7 when treated with ASK2 and then exposed to *K. pneumoniae* cells, showed a significant increase (0.82-fold) in IL-12 expression and similar response was observed with RAW 264.7 exposed to ASK2 opsonized *K. pneumoniae* (1.04-fold).

**Figure 9:**
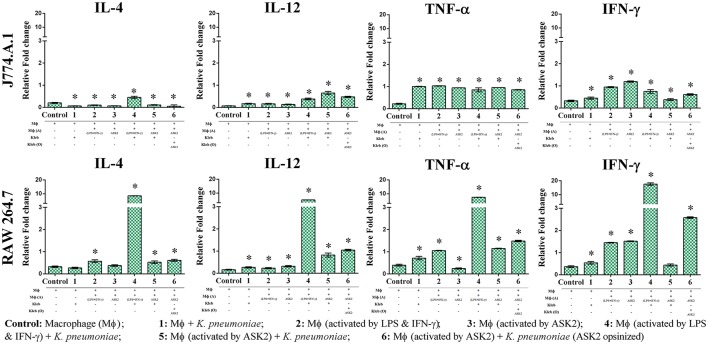
Immunomodulatory potentials of ASK2. Evaluation of cytokine expression in macrophage (MΦ) in the presence of heat killed *K. pneumoniae* (Kleb), Lipoplolysaccaride (LPS) with Interferon gamma (IFN-γ) and ASK2. IL-4, IL-12, TNF-α, and IFN-γ gene expression presented in term of relative fold change compared to GAPDH gene expression. One way ANOVA followed by multiple comparison tests were performed. ^*^ represents significant fold increase (*p* < 0.05).

In case of TNF-α gene expression in J774.A.1 there was a little variation seen across the treatment groups, where in the presence of ASK2 opsonized *K. pneumoniae*, it showed a slight enhancement in TNF-α gene expression. However, all the levels were significantly higher when compared to control cells (Figure 9; Supplementary File 5). Interestingly TNF-α gene expression was inhibited in RAW 264.7 that were treated with ASK2 alone (0.25-fold) where as in all other treatment groups, TNF-α gene expression was significantly higher when compared to untreated RAW264.7.

As expected LPS treatment of J774.A.1 resulted in significant increase in IFN-γ gene expression (0.95-fold) when compared to control (0.33-fold). This was not the case with J774.A.1 exposed to *K. pneumoniae* (0.45-fold) or J774.A.1 first treated with ASK2 and then exposed to *K. pneumoniae* (0.38-fold). However, J774.A.1 treated with ASK2 alone showed high level of IFN-γ gene expression (1.19-fold), whereas, when cells were exposed to ASK2 opsonized *K. pneumoniae* there was only a slight increase in IFN-γ gene expression (0.61-fold). In contrast, IFN-γ gene expression in RAW264.7 macrophages was observed to be high in case of cells treated with LPS (1.45-fold), ASK2 (1.52-fold), ASK2 opsonized (2.58-fold), or LPS + *K. pneumoniae* (17.59-fold). It was observed that when RAW264.7 macrophages were activated with LPS + IFN-γ and then exposed to unopsonized *K. pneumoniae*, there was a dramatic increase in the expression of all cytokines analyzed in this study. The increase was higher than that observed with any other treatment groups. In this case, IL-4 was found to be elevated by about 8-fold, IL-12 by 5-fold, TNF-α by 7-fold and as expected IFN-γ was elevated by 17-fold. These results clearly show the potency of LPS + IFN-γ in stimulating macrophage cytokine gene expression. However, what was surprising was the elevated levels of antiinflammatory IL-4 (8-fold), when compared to all other treatment groups. Whereas when IL-4 response was compared with that of ASK2 treated samples, IL-4 levels in general were observed to be very low (Figure 9). Thus, ASK2 appears to be preferentially skewing the cytokine expression.

## Discussion

Majority of the UTIs are associated with microbial colonization on indwelling urinary catheters, hence reduction of biofilm formation is a critical need to prevent CAUTI (Djeribi et al., 2012; Soto, 2014). In the present study, antibiofilm property of ASK2 obtained from *Streptomyces* sp. ASK2 was evaluated against clinical and reference strains of *K. pneumoniae*. Biofilm formation were substantially inhibited by 0.5x MIC of ASK2 with reduction of 70% for both the strains on catheter and coverslip (Figure 2). Some of the previous studies have also demonstrated the biofilm inhibitory property of various other antimicrobial agents at sub-MICs levels, (Starner et al., 2008) while others have demonstrated the growth of biofilm at sub-MICs (Dong et al., 2012). In our case, sub-MICs of ASK2 prevented *K. pneumoniae* biofilm formation suggesting that ASK2 does not trigger (or inhibits) the expression of certain virulence genes that favor biofilm formation. For example sub-MICs of ampicillin increased biofilm growth of *Staphylococcus saprophyticus*, similarly ciprofloxacin promoted biofilm of *S. saprophyticus* and *E. coli* UTI8 (Stoitsova et al., 2016). From previous reports we understood that effect of sub-MICs on biofilm varied with type of antibiotics and bacterial species (Stoitsova et al., 2016).

To further evaluate the ability of ASK2 to eradicate biofilm, different concentrations of ASK2 were used to treat 3 day old biofilm. 15-fold higher MIC were required to eradicate 75% biofilm of both the strains (Figure 3). It was observed that ESBL producing strains required 2–4 times higher MIC and 4–8 times higher corresponding MBEC values (Chaudhary and Payasi, 2012a). The MICs and MBECs of certain antibacterial agents for *K. pneumoniae* clinical isolates exhibited a similar fold increase in MIC, for example, 8x higher than their corresponding MICs of piperacillin plus tazobactam, ampicillin plus clavulanic acid, imipenem plus cilastatin, and cefoperazone plus sulbactam were found to be effective in eradicating *K. pneumoniae* biofilm. Results from the previous studies have clearly shown that, MBEC values are higher by 3–8 times in the presence of beta-lactamase inhibitor, whereas in case of antibiotics without beta-lactamase inhibitors combination, the MBEC values were higher by even 32 times. For instance, 32x MIC of meropenem were found to be significant to eradicate *K. pneumoniae* biofilm (Chaudhary and Payasi, 2012b). However, ASK2 was effective in eradicating 75% of biofilm at 15x higher MIC in the absence of beta-lactamase inhibitor. Though ASK2 has cytotoxic effect at 15x MIC (Figure 6), we believe that combinations with beta-lactamase inhibitor would significantly reduce the concentration as well as its cytotoxicity. Our results suggest that ASK2 can disperse and act on the preformed *K. pneumoniae* biofilm. However, antibiotic penetration alone is not involved in biofilm recalcitrance. For example antibiotics such as fluoroquinolones, rifampin, and ampicillin penetrate well through the biofilm matrix, but they failed to eradicate biofilm (Lebeaux et al., 2014) because the mechanism involved in biofilm recalcitrance is multi factorial. When we compared the effect of ASK2 against clinical and reference strain, we noticed a negligible variation in antibiofilm activity between them. Antibiofilm activity was further confirmed using CLSM and fluorescent microscopy (Figures 4, 5). A clear reduction in capsule thickness in treated samples, points to the fact that the antibiofilm activity of ASK2 might also interfere with adhesion mechanism of *K. pneumoniae*. Thus, ASK2 has a good potential to be used as a novel antibiofilm compound for the prevention and treatment of catheter related biofilm infections. Further investigations of genes involved in biofilm may lead to the identification of the mechanism of action of ASK2 on *K. pneumoniae* biofilm.

Having shown the antibiofilm activity of ASK2, we attempted to unravel its potential as an immunomodulant. The recognition and subsequent phagocytosis of the target microbes by macrophage constitutes an effective way of eliminating invading pathogens (Diago-Navarro et al., 2017). Opsonization of target is thus an essential attribute (Marshall et al., 2016) that can significantly increase phagocytosis.

It is evident from our results that both macrophages had more or less similar responses in terms of phagocytosis and cytokine production. The reason we used J774.A.1 and RAW 264.7 was because they have a predisposition to Th2 response (Mukherjee et al., 2009) and we wanted to check whether there is a significant proinflammatory modulation of macrophages by ASK2. However, comparatively RAW264.7 showed higher cytokine levels as well as phagocytic response when compared to J774.A.1 cells. In case of phagocytosis ASK2 proved to be a potent opsonin and was able to significantly enhance the phagocytic activity of macrophages which were not previously activated by LPS and IFN-γ (Figure 7). This shows the potential of ASK2 to function as an opsonin against *K. pneumoniae* which enables better recognition by macrophages. Indeed studies have demonstrated the opsonin effect of LPS mediated pulmonary surfactant protein-D against *K. pneumoniae* and its recognition by macrophage glycoprotein receptor (Holmskov et al., 1999; van Rozendaal et al., 2000; Ofek et al., 2001). LPS alone or as part of a whole bacterium forms an important signal to macrophages that activates signaling through TLR4 (Mukherjee et al., 2009). One of the consequence of TLR4 activation is the secretion of cytokines by macrophages which can result in further activation of macrophages but this process also contributes to the recruitment of naive T cells (Luster, 2002; Tesar et al., 2004). This is particularly relevant since LPS has been demonstrated to produce both proinflammatory (Lafleur et al., 2001) and antiinflammatory cytokine (Mukherjee et al., 2009) responses from macrophages. Apart from this, the reduction in capsule thickness induced by ASK2 (Figure 5) could also aid in enhanced phagocytic uptake, since a thicker capsule is associated with bacterial resistance.

Pretreatment of macrophages with ASK2 or opsonization of *K. pneumoniae* with ASK2 resulted in an enhanced proinflammatory cytokine gene expression. This was as expected and in case of IL-12 (both macrophages), TNF-α and IFN-γ (RAW 264.7), the response was much better when compared to LPS alone or *K. pneumoniae* alone treated cells. However, what was totally unexpected was the response of both macrophages to pretreatment with ASK2 or to ASK2-opsonized *K. pneumoniae*, where IL-4 levels were inhibited. These results indicate that in both macrophages, ASK2 has a stimulating effect on IL-12, IFN-γ, and TNF-α only when it was used to pretreat (= opsonization) *K. pneumoniae* before exposing them to macrophages except in the case of J774.A.1, in which IFN-γ gene expression was marginal. On the other hand, ASK2 alone was stimulatory only for IFN-γ gene expression in both the macrophages. The results not only suggest the opsonic role of ASK2 but also show its potential in modulating proinflammatory cytokines secreted by macrophages.

**Figure 10:**
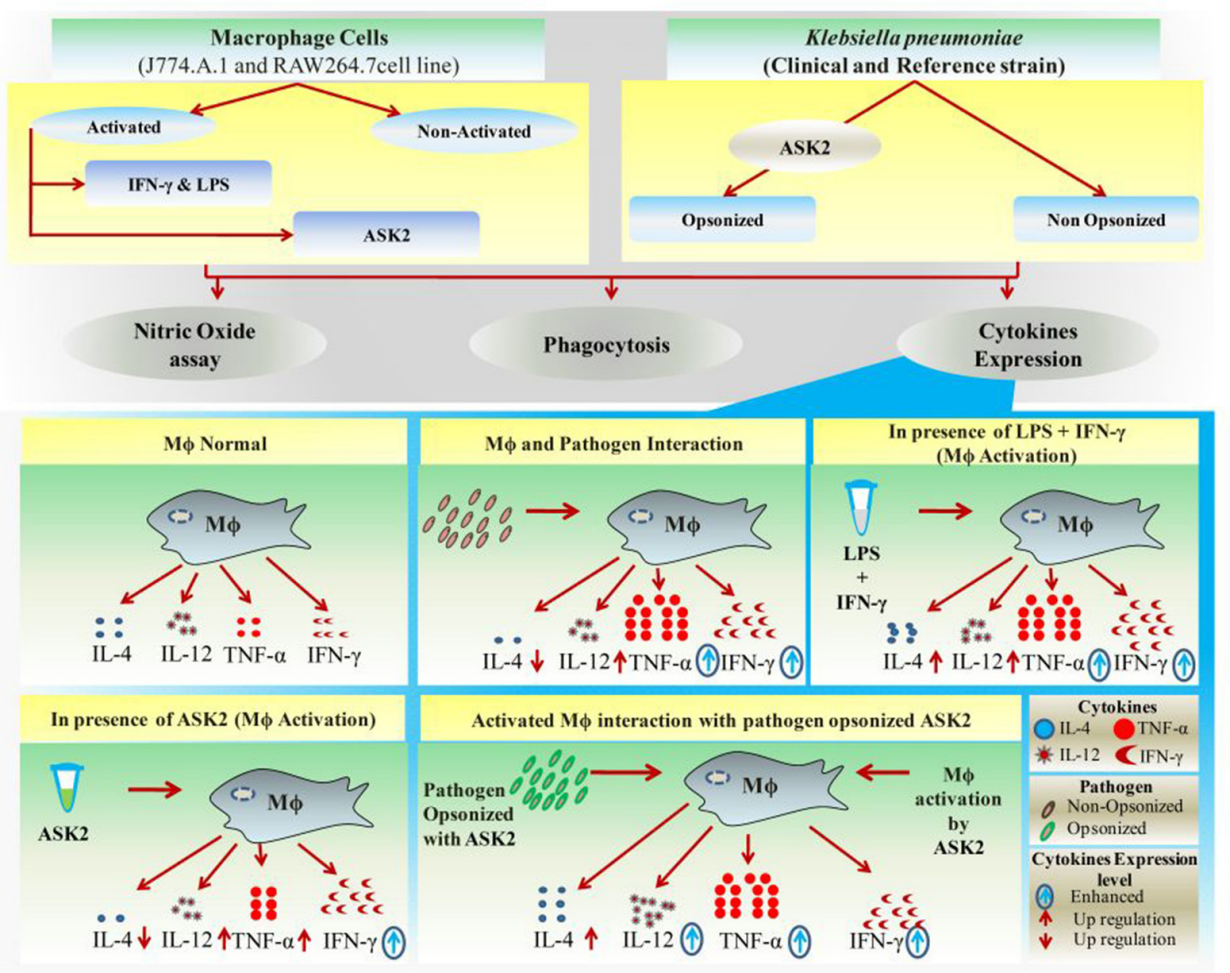
Graphical representation. Significant effects of ASK2 on macrophage to enhance phagocytosis and modulate cytokine production during host pathogen interactions.

Other previous studies too supports our contention on ASK2 and IL-4 gene expression, wherein the authors have demonstrated that immune complexes (ASK2-opsonized bacteria in this study) in a highly inflammatory environment can actually promote type II activation of macrophages leading to Th2 cytokines, such as IL-4 (Sutterwala et al., 1998; Camille et al., 2012). Such a response, unfortunately, favors bacteria survival and suppresses the rapid and effective antibacterial functions of proinflammatory cytokines. Interestingly, in the presence of ASK2, IL-4 levels were observed to be very low when compared to all treatment groups. By promoting proinflammatory cytokine expression and inhibiting antiinflammatory response of macrophages (Figure 10) ASK2 can help in increasing protective immunity against invading pathogens such as *K. pneumoniae*.

## Conclusion

The *Streptomyces* derived ASK2 bioactive compound appears to possess dual activities. On one hand it is antibacterial and antibiofilm and on the other it showed *in vitro* immunomodulation by altering both phagocytic rates of macrophages as well as skewing the macrophage cytokine response toward a more proinflammatory one. However, further studies with *in vivo* models are required before the possible clinical application of ASK2 to combat persistent MDR *K. pneumoniae*.

## Author contributions

All authors listed have made a substantial, direct and intellectual contribution to the work, and approved it for publication.

### Conflict of interest statement

The authors declare that the research was conducted in the absence of any commercial or financial relationships that could be construed as a potential conflict of interest.
