# Supplementary material for: ASK2 Bioactive Compound Inhibits MDR Klebsiella pneumoniae by Antibiofilm Activity, Modulating Macrophage Cytokines and Opsonophagocytosis
Source: Front Cell Infect Microbiol. 2017 Aug 4;7:346. doi: 10.3389/fcimb.2017.00346 (PMC5543099; doi:10.3389/fcimb.2017.00346)
Supplement: Supplementary file 1 [file Table1.DOCX]

**Supplementary File 1:** Experimental Group details for Phagocytosis assay

| **Groups** | **J774.A.1 & Raw 264.7** | **Clinical strain** |
| --- | --- | --- |
|  | **Macrophage (Mφ)** | ***Klebsiella* (Kleb.)** |
|  |  |  |
| **Group 1**  **(Control)** | Mφ  (Untreated) | - |
| **Group 2**  **(Host Pathogen Interaction)** | Mφ  (Untreated) | Kleb.  (Untreated) |
| **Group 3**  **(Mφ activated + pathogen)** | Mφ  (LPS 3μg/ml + IFN-γ 100 picomol) | Kleb.  (Untreated) |
| **Group 4**  **(Mφ activated + Opsonized pathogen)** | Mφ  (Untreated) | Kleb.  (Treated with 0.5x MIC) |
| **Group 5**  **(Mφ activated + Opsonized pathogen)** | Mφ  (Untreated) | Kleb.  (Treated with 1x MIC) |
| **Group 6**  **(Mφ activated + Opsonized pathogen)** | Mφ  (LPS + IFN-γ) | Kleb.  (Treated with 0.5x MIC) |
| **Group 7**  **(Mφ activated + Opsonized pathogen)** | Mφ  (LPS + IFN-γ) | Kleb.  (Treated with 1x MIC) |
