# Supplementary material for: ASK2 Bioactive Compound Inhibits MDR Klebsiella pneumoniae by Antibiofilm Activity, Modulating Macrophage Cytokines and Opsonophagocytosis
Source: Front Cell Infect Microbiol. 2017 Aug 4;7:346. doi: 10.3389/fcimb.2017.00346 (PMC5543099; doi:10.3389/fcimb.2017.00346)
Supplement: Supplementary file 2 [file Table2.DOCX]

**Supplementary File 2: Normalization of Cytokine expression**

To find the relative gene expression, housekeeping gene DNA band intensity peak area (HK_area_) value for all groups was considered as 1 fold expression and relative to that other target gene DNA band intensity peak area (Target_area_) was calculated by following equation:

For housekeeping gene expression:

Fold change = (HK_area_)/ (HK_area_)

For target gene expression

Fold change = (Target_area_)/ (HK_area_)

Normalization: For normalization target gene fold expression (T_target_GFE) subtract from housekeeping gene fold expression (HGFE); (A = T_target_GFE -HGFE), followed by treatment group fold expression (A_treatment_GFE) subtract from control group fold expression (A_control_GFE); (B=A_treatment_GFE- A_control_GFE). Calculated value B used for analysis and graph preparation.
