# Supplementary material for: ASK2 Bioactive Compound Inhibits MDR Klebsiella pneumoniae by Antibiofilm Activity, Modulating Macrophage Cytokines and Opsonophagocytosis
Source: Front Cell Infect Microbiol. 2017 Aug 4;7:346. doi: 10.3389/fcimb.2017.00346 (PMC5543099; doi:10.3389/fcimb.2017.00346)
Supplement: Supplementary file 3 [file Table3.DOCX]

**Supplementary File 3:** Cell size, capsule size and capsule thickness of *Klebsiella pneumoniae* clinical and reference strain on coverslip and catheter analyze by fluorescence microscopy

| **Cell size (in µm)** | | | | | | | | | | | |
| --- | --- | --- | --- | --- | --- | --- | --- | --- | --- | --- | --- |
|  | **Clinical** | **Clinical** | | **Clinical** | **Clinical** | | **MCC** | **MCC** | | **MCC** | **MCC** |
|  | **Coverslip** | **Coverslip** | | **Catheter** | **Catheter** | | **Coverslip** | **Coverslip** | | **Catheter** | **Catheter** |
|  | **Avg** | **SD** | | **Avg** | **SD** | | **Avg** | **SD** | | **Avg** | **SD** |
| **Control** | **1.787** | **0.256** | | **2.182** | **0.253** | | **2.015** | **0.265** | | **2.079** | **0.230** |
| **0.5X MIC** | **2.440** | **0.204** | | **2.003** | **0.248** | | **2.028** | **0.265** | | **2.143** | **0.417** |
| **15X MIC** | **2.097** | **0.543** | | **1.865** | **0.355** | | **1.435** | **0.247** | | **1.696** | **0.355** |
|  |  |  | |  |  | |  |  | |  |  |
| **Cell + Capsule Size (in µm)** | | | | | | | | | | | |
|  | **Clinical** | | **Clinical** | **Clinical** | | **Clinical** | **MCC** | | **MCC** | **MCC** | **MCC** |
|  | **Coverslip** | | **Coverslip** | **Catheter** | | **Catheter** | **Coverslip** | | **Coverslip** | **Catheter** | **Catheter** |
|  | **Avg** | | **SD** | **Avg** | | **SD** | **Avg** | | **SD** | **Avg** | **SD** |
| **Control** | **4.029** | | **0.941** | **3.677** | | **0.084** | **4.362** | | **0.088** | **3.712** | **0.077** |
| **0.5X MIC** | **3.513 (12.8)** | | **0.458** | **2.793 (24.04)** | | **0.316** | **3.099 (28.95)** | | **0.440** | **3.061(17.53)** | **0.375** |
| **15X MIC** | **3.077 (23.62)** | | **0.605** | **2.839 (22.79)** | | **0.516** | **2.353 (46.05)** | | **0.289** | **2.659(28.36)** | **0.711** |
|  |  | |  |  | |  |  | |  |  |  |
| **Capsule Thickness (in µm)** | | | | | | | | | | | |
|  | **Clinical** | | **Clinical** | **Clinical** | **Clinical** | | **MCC** | **MCC** | | **MCC** | **MCC** |
|  | **Coverslip** | | **Coverslip** | **Catheter** | **Catheter** | | **Coverslip** | **Coverslip** | | **Catheter** | **Catheter** |
|  | **Avg** | | **SD** | **Avg** | **SD** | | **Avg** | **SD** | | **Avg** | **SD** |
| **Control** | **2.242** | | **0.689** | **1.495** | **0.337** | | **2.347** | **0.353** | | **1.633** | **0.306** |
| **0.5X MIC** | **1.073 (52.14)** | | **0.434** | **0.791(47.09)** | **0.290** | | **1.071(54.36)** | **0.273** | | **0.918(43.78)** | **0.042** |
| **15X MIC** | **0.980 (56.28)** | | **0.302** | **0.974(34.84)** | **0.214** | | **0.918(60.88)** | **0.138** | | **0.963(41.02)** | **0.367** |
