# Supplementary material for: ASK2 Bioactive Compound Inhibits MDR Klebsiella pneumoniae by Antibiofilm Activity, Modulating Macrophage Cytokines and Opsonophagocytosis
Source: Front Cell Infect Microbiol. 2017 Aug 4;7:346. doi: 10.3389/fcimb.2017.00346 (PMC5543099; doi:10.3389/fcimb.2017.00346)
Supplement: Supplementary file 4 [file Table4.DOCX]

**Supplementary File 4:** Statistical analysis of phagocytosis assay

| **1** | **J774.A.1** |  |  |  |  |  |
| --- | --- | --- | --- | --- | --- | --- |
|  | **ANOVA summary** |  |  |  |  |  |
|  | F | 59.92 |  |  |  |  |
|  | P value | < 0.0001 |  |  |  |  |
|  | P value summary | **** |  |  |  |  |
|  | Are differences among means statistically significant? (P < 0.05) | Yes |  |  |  |  |
|  | R square | 0.9615 |  |  |  |  |
|  |  |  |  |  |  |  |
|  | **ANOVA table** | **SS** | **DF** | **MS** | **F (DFn, DFd)** | **P value** |
|  | Treatment (between columns) | 4090 | 5 | 818.1 | F (5, 12) = 59.92 | P < 0.0001 |
|  | Residual (within columns) | 163.8 | 12 | 13.65 |  |  |
|  | Total | 4254 | 17 |  |  |  |
|  |  |  |  |  |  |  |
|  | **Tukey's multiple comparisons test** | **Mean Diff.** | **95% CI of diff.** | **Significant?** | **Summary** | **Adjusted P Value** |
|  | Mϕ(A) + Kleb ***vs.*** Mϕ Kleb | 13.62 | 3.490 to 23.76 | Yes | ** | 0.0071 |
|  | Mϕ Kleb (O1) ***vs.*** Mϕ Kleb | 20.36 | 10.23 to 30.50 | Yes | *** | 0.0002 |
|  | Mϕ Kleb (O2) ***vs.*** Mϕ Kleb | 25.08 | 14.95 to 35.22 | Yes | **** | < 0.0001 |
|  | Mϕ(A) + Kleb (O1) ***vs.*** Mϕ Kleb | 39.41 | 29.28 to 49.55 | Yes | **** | < 0.0001 |
|  | Mϕ(A) + Kleb (O2) ***vs.*** Mϕ Kleb | 44.69 | 34.55 to 54.82 | Yes | **** | < 0.0001 |
|  | Mϕ Kleb (O1) ***vs.*** Mϕ(A) + Kleb | 6.738 | -3.396 to 16.87 | No | ns | 0.2912 |
|  | Mϕ Kleb (O2) ***vs.*** Mϕ(A) + Kleb | 11.46 | 1.324 to 21.59 | Yes | * | 0.0239 |
|  | Mϕ(A) + Kleb (O1) ***vs.*** Mϕ(A) + Kleb | 25.79 | 15.66 to 35.92 | Yes | **** | < 0.0001 |
|  | Mϕ(A) + Kleb (O2) ***vs.*** Mϕ(A) + Kleb | 31.06 | 20.93 to 41.20 | Yes | **** | < 0.0001 |
|  | Mϕ Kleb (O2) ***vs.*** Mϕ Kleb (O1) | 4.72 | -5.414 to 14.85 | No | ns | 0.6338 |
|  | Mϕ(A) + Kleb (O1) ***vs.*** Mϕ Kleb (O1) | 19.05 | 8.920 to 29.19 | Yes | *** | 0.0004 |
|  | Mϕ(A) + Kleb (O2) ***vs.*** Mϕ Kleb (O1) | 24.32 | 14.19 to 34.46 | Yes | **** | < 0.0001 |
|  | Mϕ(A) + Kleb (O1) ***vs.*** Mϕ Kleb (O2) | 14.33 | 4.200 to 24.47 | Yes | ** | 0.0049 |
|  | Mϕ(A) + Kleb (O2) ***vs.*** Mϕ Kleb (O2) | 19.6 | 9.470 to 29.74 | Yes | *** | 0.0003 |
|  | Mϕ(A) + Kleb (O2) ***vs.*** Mϕ(A) + Kleb (O1) | 5.27 | -4.863 to 15.40 | No | ns | 0.5297 |
|  |  |  |  |  |  |  |

| **2** | **Raw264.7** |  |  |  |  |  |
| --- | --- | --- | --- | --- | --- | --- |
|  | **ANOVA summary** |  |  |  |  |  |
|  | F | 89.88 |  |  |  |  |
|  | P value | < 0.0001 |  |  |  |  |
|  | P value summary | **** |  |  |  |  |
|  | Are differences among means statistically significant? (P < 0.05) | Yes |  |  |  |  |
|  | R square | 0.974 |  |  |  |  |
|  |  |  |  |  |  |  |
|  | **ANOVA table** | **SS** | **DF** | **MS** | **F (DFn, DFd)** | **P value** |
|  | Treatment (between columns) | 5529 | 5 | 1106 | F (5, 12) = 89.88 | P < 0.0001 |
|  | Residual (within columns) | 147.6 | 12 | 12.3 |  |  |
|  | Total | 5677 | 17 |  |  |  |
|  |  |  |  |  |  |  |
|  | **Tukey's multiple comparisons test** | **Mean Diff.** | **95% CI of diff.** | **Significant?** | **Summary** | **Adjusted P Value** |
|  | Mϕ(A) + Kleb ***vs.*** Mϕ Kleb | 15.4 | 5.784 to 25.02 | Yes | ** | 0.0018 |
|  | Mϕ Kleb (O1) ***vs.*** Mϕ Kleb | 23.39 | 13.77 to 33.01 | Yes | **** | < 0.0001 |
|  | Mϕ Kleb (O2) ***vs.*** Mϕ Kleb | 32 | 22.38 to 41.62 | Yes | **** | < 0.0001 |
|  | Mϕ(A) + Kleb (O1) ***vs.*** Mϕ Kleb | 44.74 | 35.12 to 54.36 | Yes | **** | < 0.0001 |
|  | Mϕ(A) + Kleb (O2) ***vs.*** Mϕ Kleb | 52.15 | 42.53 to 61.77 | Yes | **** | < 0.0001 |
|  | Mϕ Kleb (O1) ***vs.*** Mϕ(A) + Kleb | 7.989 | -1.631 to 17.61 | No | ns | 0.127 |
|  | Mϕ Kleb (O2) ***vs.*** Mϕ(A) + Kleb | 16.59 | 6.973 to 26.21 | Yes | *** | 0.0009 |
|  | Mϕ(A) + Kleb (O1) ***vs.*** Mϕ(A) + Kleb | 29.33 | 19.71 to 38.95 | Yes | **** | < 0.0001 |
|  | Mϕ(A) + Kleb (O2) ***vs.*** Mϕ(A) + Kleb | 36.74 | 27.12 to 46.36 | Yes | **** | < 0.0001 |
|  | Mϕ Kleb (O2) ***vs.*** Mϕ Kleb (O1) | 8.604 | -1.016 to 18.22 | No | ns | 0.0899 |
|  | Mϕ(A) + Kleb (O1) ***vs.*** Mϕ Kleb (O1) | 21.34 | 11.72 to 30.96 | Yes | **** | < 0.0001 |
|  | Mϕ(A) + Kleb (O2) ***vs.*** Mϕ Kleb (O1) | 28.75 | 19.13 to 38.37 | Yes | **** | < 0.0001 |
|  | Mϕ(A) + Kleb (O1) ***vs.*** Mϕ Kleb (O2) | 12.74 | 3.119 to 22.36 | Yes | ** | 0.008 |
|  | Mϕ(A) + Kleb (O2) ***vs.*** Mϕ Kleb (O2) | 20.15 | 10.53 to 29.77 | Yes | *** | 0.0002 |
|  | Mϕ(A) + Kleb (O2) ***vs.*** Mϕ(A) + Kleb (O1) | 7.411 | -2.209 to 17.03 | No | ns | 0.1739 |
