# Supplementary material for: ASK2 Bioactive Compound Inhibits MDR Klebsiella pneumoniae by Antibiofilm Activity, Modulating Macrophage Cytokines and Opsonophagocytosis
Source: Front Cell Infect Microbiol. 2017 Aug 4;7:346. doi: 10.3389/fcimb.2017.00346 (PMC5543099; doi:10.3389/fcimb.2017.00346)
Supplement: Supplementary file 5 [file Table5.DOCX]

**Supplementary File 5:** Statistics Analysis for Gene expression of IL-4, IL-12, TNF-α, IFN-γ in J774.A.1 and Raw264.7

| **Conditions** | **1** | **2** | **3** | **4** | **5** | **6** |
| --- | --- | --- | --- | --- | --- | --- |
| **Mɸ** | **+** | **+** | **+** | **+** | **+** | **+** |
| **Mɸ (A)** | **-** | **+** | **+** | **+** | **+** | **+** |
|  |  | LPS + IFN-γ | ASK2 | LPS + IFN-γ | ASK2 | ASK2 |
| **Kleb** | **+** | **-** | **-** | **+** | **+** | **-** |
| **Kleb (O)** | **-** | **-** | **-** | **-** | **-** | **+** |
|  |  |  |  |  |  | ASK2 |

| **1.** | **J774.A.1** |  |  |  |  |  |
| --- | --- | --- | --- | --- | --- | --- |
| **1.1.** | **IL-4** |  |  |  |  |  |
|  | **ANOVA summary** |  |  |  |  |  |
|  | F | 21.85 |  |  |  |  |
|  | P value | 0.0009 |  |  |  |  |
|  | P value summary | *** |  |  |  |  |
|  | Are differences among means statistically significant? (P < 0.05) | Yes |  |  |  |  |
|  | R square | 0.9479 |  |  |  |  |
|  |  |  |  |  |  |  |
|  | **ANOVA table** | **SS** | **DF** | **MS** | **F (DFn, DFd)** | **P value** |
|  | Treatment (between columns) | 0.2337 | 5 | 0.04675 | F (5, 6) = 21.85 | P = 0.0009 |
|  | Residual (within columns) | 0.01284 | 6 | 0.00214 |  |  |
|  | Total | 0.2466 | 11 |  |  |  |
|  |  |  |  |  |  |  |
|  | **Tukey's multiple comparisons test** | **Mean Diff.** | **95% CI of diff.** | **Significant?** | **Summary** | **Adjusted P Value** |
|  | 2 vs. 1 | 0.035 | -0.1491 to 0.2191 | No | ns | 0.9658 |
|  | 3 vs. 1 | 0.0027 | -0.1814 to 0.1868 | No | ns | > 0.9999 |
|  | 4 vs. 1 | 0.3839 | 0.1998 to 0.5679 | Yes | ** | 0.0013 |
|  | 5 vs. 1 | 0.0426 | -0.1415 to 0.2267 | No | ns | 0.9272 |
|  | 6 vs. 1 | -0.01435 | -0.1984 to 0.1697 | No | ns | 0.9994 |
|  | 3 vs. 2 | -0.0323 | -0.2164 to 0.1518 | No | ns | 0.9754 |
|  | 4 vs. 2 | 0.3489 | 0.1648 to 0.5329 | Yes | ** | 0.0022 |
|  | 5 vs. 2 | 0.0076 | -0.1765 to 0.1917 | No | ns | > 0.9999 |
|  | 6 vs. 2 | -0.04935 | -0.2334 to 0.1347 | No | ns | 0.8784 |
|  | 4 vs. 3 | 0.3812 | 0.1971 to 0.5652 | Yes | ** | 0.0014 |
|  | 5 vs. 3 | 0.0399 | -0.1442 to 0.2240 | No | ns | 0.943 |
|  | 6 vs. 3 | -0.01705 | -0.2011 to 0.1670 | No | ns | 0.9986 |
|  | 5 vs. 4 | -0.3413 | -0.5253 to -0.1572 | Yes | ** | 0.0025 |
|  | 6 vs. 4 | -0.3982 | -0.5823 to -0.2141 | Yes | ** | 0.0011 |
|  | 6 vs. 5 | -0.05695 | -0.2410 to 0.1271 | No | ns | 0.8095 |
|  |  |  |  |  |  |  |
| **1.2.** | **IL-12** |  |  |  |  |  |
|  | **ANOVA summary** |  |  |  |  |  |
|  | F | 32.11 |  |  |  |  |
|  | P value | 0.0003 |  |  |  |  |
|  | P value summary | *** |  |  |  |  |
|  | Are differences among means statistically significant? (P < 0.05) | Yes |  |  |  |  |
|  | R square | 0.964 |  |  |  |  |
|  |  |  |  |  |  |  |
|  | **ANOVA table** | **SS** | **DF** | **MS** | **F (DFn, DFd)** | **P value** |
|  | Treatment (between columns) | 0.4369 | 5 | 0.08738 | F (5, 6) = 32.11 | P = 0.0003 |
|  | Residual (within columns) | 0.01633 | 6 | 0.002721 |  |  |
|  | Total | 0.4532 | 11 |  |  |  |
|  |  |  |  |  |  |  |
|  | **Tukey's multiple comparisons test** | **Mean Diff.** | **95% CI of diff.** | **Significant?** | **Summary** | **Adjusted P Value** |
|  | 2 vs. 1 | -0.00415 | -0.2118 to 0.2035 | No | ns | > 0.9999 |
|  | 3 vs. 1 | -0.03255 | -0.2402 to 0.1751 | No | ns | 0.9847 |
|  | 4 vs. 1 | 0.2134 | 0.005790 to 0.4210 | Yes | * | 0.0445 |
|  | 5 vs. 1 | 0.4826 | 0.2749 to 0.6902 | Yes | *** | 0.0007 |
|  | 6 vs. 1 | 0.3082 | 0.1005 to 0.5158 | Yes | ** | 0.0079 |
|  | 3 vs. 2 | -0.0284 | -0.2360 to 0.1792 | No | ns | 0.9916 |
|  | 4 vs. 2 | 0.2176 | 0.009940 to 0.4252 | Yes | * | 0.0409 |
|  | 5 vs. 2 | 0.4867 | 0.2791 to 0.6943 | Yes | *** | 0.0007 |
|  | 6 vs. 2 | 0.3123 | 0.1047 to 0.5199 | Yes | ** | 0.0074 |
|  | 4 vs. 3 | 0.246 | 0.03834 to 0.4536 | Yes | * | 0.0236 |
|  | 5 vs. 3 | 0.5151 | 0.3075 to 0.7227 | Yes | *** | 0.0005 |
|  | 6 vs. 3 | 0.3407 | 0.1331 to 0.5483 | Yes | ** | 0.0047 |
|  | 5 vs. 4 | 0.2692 | 0.06154 to 0.4768 | Yes | * | 0.0154 |
|  | 6 vs. 4 | 0.09475 | -0.1129 to 0.3024 | No | ns | 0.5182 |
|  | 6 vs. 5 | -0.1744 | -0.3820 to 0.03321 | No | ns | 0.0999 |
|  |  |  |  |  |  |  |
| **1.3.** | **TNF-α** |  |  |  |  |  |
|  | **ANOVA summary** |  |  |  |  |  |
|  | F | 3.767 |  |  |  |  |
|  | P value | 0.0686 |  |  |  |  |
|  | P value summary | Ns |  |  |  |  |
|  | Are differences among means statistically significant? (P < 0.05) | No |  |  |  |  |
|  | R square | 0.7584 |  |  |  |  |
|  |  |  |  |  |  |  |
|  | **ANOVA table** | **SS** | **DF** | **MS** | **F (DFn, DFd)** | **P value** |
|  | Treatment (between columns) | 0.05483 | 5 | 0.01097 | F (5, 6) = 3.767 | P = 0.0686 |
|  | Residual (within columns) | 0.01746 | 6 | 0.002911 |  |  |
|  | Total | 0.07229 | 11 |  |  |  |
|  |  |  |  |  |  |  |
|  | **Tukey's multiple comparisons test** | **Mean Diff.** | **95% CI of diff.** | **Significant?** | **Summary** | **Adjusted P Value** |
|  | 2 vs. 1 | 0.0293 | -0.1854 to 0.2440 | No | ns | 0.9917 |
|  | 3 vs. 1 | -0.05855 | -0.2733 to 0.1562 | No | ns | 0.8714 |
|  | 4 vs. 1 | -0.1501 | -0.3648 to 0.06461 | No | ns | 0.1871 |
|  | 5 vs. 1 | -0.049 | -0.2637 to 0.1657 | No | ns | 0.9308 |
|  | 6 vs. 1 | -0.1474 | -0.3621 to 0.06731 | No | ns | 0.1979 |
|  | 3 vs. 2 | -0.08785 | -0.3026 to 0.1269 | No | ns | 0.6116 |
|  | 4 vs. 2 | -0.1794 | -0.3941 to 0.03531 | No | ns | 0.1019 |
|  | 5 vs. 2 | -0.0783 | -0.2930 to 0.1364 | No | ns | 0.7021 |
|  | 6 vs. 2 | -0.1767 | -0.3914 to 0.03801 | No | ns | 0.1077 |
|  | 4 vs. 3 | -0.09155 | -0.3063 to 0.1232 | No | ns | 0.5769 |
|  | 5 vs. 3 | 0.00955 | -0.2052 to 0.2243 | No | ns | > 0.9999 |
|  | 6 vs. 3 | -0.08885 | -0.3036 to 0.1259 | No | ns | 0.6021 |
|  | 5 vs. 4 | 0.1011 | -0.1136 to 0.3158 | No | ns | 0.491 |
|  | 6 vs. 4 | 0.0027 | -0.2120 to 0.2174 | No | ns | > 0.9999 |
|  | 6 vs. 5 | -0.0984 | -0.3131 to 0.1163 | No | ns | 0.5146 |
|  |  |  |  |  |  |  |
| **1.4.** | **IFN-γ** |  |  |  |  |  |
|  | **ANOVA summary** |  |  |  |  |  |
|  | F | 42.95 |  |  |  |  |
|  | P value | 0.0001 |  |  |  |  |
|  | P value summary | *** |  |  |  |  |
|  | Are differences among means statistically significant? (P < 0.05) | Yes |  |  |  |  |
|  | R square | 0.9728 |  |  |  |  |
|  |  |  |  |  |  |  |
|  | **ANOVA table** | **SS** | **DF** | **MS** | **F (DFn, DFd)** | **P value** |
|  | Treatment (between columns) | 0.9499 | 5 | 0.19 | F (5, 6) = 42.95 | P = 0.0001 |
|  | Residual (within columns) | 0.02654 | 6 | 0.004423 |  |  |
|  | Total | 0.9765 | 11 |  |  |  |
|  |  |  |  |  |  |  |
|  | **Tukey's multiple comparisons test** | **Mean Diff.** | **95% CI of diff.** | **Significant?** | **Summary** | **Adjusted P Value** |
|  | 2 vs. 1 | 0.4966 | 0.2319 to 0.7612 | Yes | ** | 0.0023 |
|  | 3 vs. 1 | 0.7441 | 0.4794 to 1.009 | Yes | *** | 0.0002 |
|  | 4 vs. 1 | 0.3007 | 0.03602 to 0.5654 | Yes | * | 0.0286 |
|  | 5 vs. 1 | -0.06705 | -0.3317 to 0.1976 | No | ns | 0.8996 |
|  | 6 vs. 1 | 0.1627 | -0.1020 to 0.4274 | No | ns | 0.2716 |
|  | 3 vs. 2 | 0.2475 | -0.01718 to 0.5122 | No | ns | 0.0659 |
|  | 4 vs. 2 | -0.1959 | -0.4605 to 0.06883 | No | ns | 0.1559 |
|  | 5 vs. 2 | -0.5636 | -0.8283 to -0.2989 | Yes | ** | 0.0012 |
|  | 6 vs. 2 | -0.3339 | -0.5985 to -0.06917 | Yes | * | 0.0176 |
|  | 4 vs. 3 | -0.4434 | -0.7080 to -0.1787 | Yes | ** | 0.0042 |
|  | 5 vs. 3 | -0.8111 | -1.076 to -0.5464 | Yes | *** | 0.0002 |
|  | 6 vs. 3 | -0.5814 | -0.8460 to -0.3167 | Yes | *** | 0.001 |
|  | 5 vs. 4 | -0.3678 | -0.6324 to -0.1031 | Yes | * | 0.011 |
|  | 6 vs. 4 | -0.138 | -0.4027 to 0.1267 | No | ns | 0.4027 |
|  | 6 vs. 5 | 0.2298 | -0.03493 to 0.4944 | No | ns | 0.0883 |

| **2** | **Raw 264.7** |  |  |  |  |  |
| --- | --- | --- | --- | --- | --- | --- |
| **2.1.** | **IL-4** |  |  |  |  |  |
|  | **ANOVA summary** |  |  |  |  |  |
|  | F | 3214 |  |  |  |  |
|  | P value | < 0.0001 |  |  |  |  |
|  | P value summary | **** |  |  |  |  |
|  | Are differences among means statistically significant? (P < 0.05) | Yes |  |  |  |  |
|  | R square | 0.9996 |  |  |  |  |
|  |  |  |  |  |  |  |
|  | **ANOVA table** | **SS** | **DF** | **MS** | **F (DFn, DFd)** | **P value** |
|  | Treatment (between columns) | 107.2 | 5 | 21.43 | F (5, 6) = 3214 | P < 0.0001 |
|  | Residual (within columns) | 0.04001 | 6 | 0.006668 |  |  |
|  | Total | 107.2 | 11 |  |  |  |
|  |  |  |  |  |  |  |
|  | **Tukey's multiple comparisons test** | **Mean Diff.** | **95% CI of diff.** | **Significant?** | **Summary** | **Adjusted P Value** |
|  | 2 vs. 1 | 0.3035 | -0.02154 to 0.6284 | No | ns | 0.0663 |
|  | 3 vs. 1 | 0.103 | -0.2220 to 0.4280 | No | ns | 0.7955 |
|  | 4 vs. 1 | 8.213 | 7.888 to 8.538 | Yes | **** | < 0.0001 |
|  | 5 vs. 1 | 0.2565 | -0.06849 to 0.5815 | No | ns | 0.1251 |
|  | 6 vs. 1 | 0.3417 | 0.01671 to 0.6667 | Yes | * | 0.0403 |
|  | 3 vs. 2 | -0.2005 | -0.5254 to 0.1245 | No | ns | 0.2691 |
|  | 4 vs. 2 | 7.91 | 7.585 to 8.235 | Yes | **** | < 0.0001 |
|  | 5 vs. 2 | -0.04695 | -0.3719 to 0.2780 | No | ns | 0.9893 |
|  | 6 vs. 2 | 0.03825 | -0.2867 to 0.3632 | No | ns | 0.9957 |
|  | 4 vs. 3 | 8.11 | 7.785 to 8.435 | Yes | **** | < 0.0001 |
|  | 5 vs. 3 | 0.1535 | -0.1715 to 0.4785 | No | ns | 0.4883 |
|  | 6 vs. 3 | 0.2387 | -0.08629 to 0.5637 | No | ns | 0.1597 |
|  | 5 vs. 4 | -7.957 | -8.282 to -7.632 | Yes | **** | < 0.0001 |
|  | 6 vs. 4 | -7.871 | -8.196 to -7.546 | Yes | **** | < 0.0001 |
|  | 6 vs. 5 | 0.0852 | -0.2398 to 0.4102 | No | ns | 0.8871 |
|  |  |  |  |  |  |  |
| **2.2.** | **IL-12** |  |  |  |  |  |
|  | **ANOVA summary** |  |  |  |  |  |
|  | F | 1397 |  |  |  |  |
|  | P value | < 0.0001 |  |  |  |  |
|  | P value summary | **** |  |  |  |  |
|  | Are differences among means statistically significant? (P < 0.05) | Yes |  |  |  |  |
|  | R square | 0.9991 |  |  |  |  |
|  |  |  |  |  |  |  |
|  | **ANOVA table** | **SS** | **DF** | **MS** | **F (DFn, DFd)** | **P value** |
|  | Treatment (between columns) | 34.8 | 5 | 6.96 | F (5, 6) = 1397 | P < 0.0001 |
|  | Residual (within columns) | 0.02989 | 6 | 0.004981 |  |  |
|  | Total | 34.83 | 11 |  |  |  |
|  |  |  |  |  |  |  |
|  | **Tukey's multiple comparisons test** | **Mean Diff.** | **95% CI of diff.** | **Significant?** | **Summary** | **Adjusted P Value** |
|  | 2 vs. 1 | -0.03405 | -0.3149 to 0.2468 | No | ns | 0.9951 |
|  | 3 vs. 1 | 0.05035 | -0.2305 to 0.3312 | No | ns | 0.9731 |
|  | 4 vs. 1 | 4.767 | 4.486 to 5.047 | Yes | **** | < 0.0001 |
|  | 5 vs. 1 | 0.5583 | 0.2774 to 0.8392 | Yes | ** | 0.0017 |
|  | 6 vs. 1 | 0.7786 | 0.4977 to 1.059 | Yes | *** | 0.0003 |
|  | 3 vs. 2 | 0.0844 | -0.1965 to 0.3653 | No | ns | 0.8253 |
|  | 4 vs. 2 | 4.801 | 4.520 to 5.081 | Yes | **** | < 0.0001 |
|  | 5 vs. 2 | 0.5924 | 0.3115 to 0.8732 | Yes | ** | 0.0012 |
|  | 6 vs. 2 | 0.8127 | 0.5318 to 1.094 | Yes | *** | 0.0002 |
|  | 4 vs. 3 | 4.716 | 4.435 to 4.997 | Yes | **** | < 0.0001 |
|  | 5 vs. 3 | 0.508 | 0.2271 to 0.7888 | Yes | ** | 0.0028 |
|  | 6 vs. 3 | 0.7283 | 0.4474 to 1.009 | Yes | *** | 0.0004 |
|  | 5 vs. 4 | -4.208 | -4.489 to -3.927 | Yes | **** | < 0.0001 |
|  | 6 vs. 4 | -3.988 | -4.269 to -3.707 | Yes | **** | < 0.0001 |
|  | 6 vs. 5 | 0.2203 | -0.06058 to 0.5012 | No | ns | 0.1279 |
|  |  |  |  |  |  |  |
| **2.3.** | **TNF-α** |  |  |  |  |  |
|  | **ANOVA summary** |  |  |  |  |  |
|  | F | 2992 |  |  |  |  |
|  | P value | < 0.0001 |  |  |  |  |
|  | P value summary | **** |  |  |  |  |
|  | Are differences among means statistically significant? (P < 0.05) | Yes |  |  |  |  |
|  | R square | 0.9996 |  |  |  |  |
|  |  |  |  |  |  |  |
|  | **ANOVA table** | **SS** | **DF** | **MS** | **F (DFn, DFd)** | **P value** |
|  | Treatment (between columns) | 72.96 | 5 | 14.59 | F (5, 6) = 2992 | P < 0.0001 |
|  | Residual (within columns) | 0.02926 | 6 | 0.004877 |  |  |
|  | Total | 72.99 | 11 |  |  |  |
|  |  |  |  |  |  |  |
|  | **Tukey's multiple comparisons test** | **Mean Diff.** | **95% CI of diff.** | **Significant?** | **Summary** | **Adjusted P Value** |
|  | 2 vs. 1 | 0.3343 | 0.05633 to 0.6122 | Yes | * | 0.022 |
|  | 3 vs. 1 | -0.4741 | -0.7520 to -0.1961 | Yes | ** | 0.0039 |
|  | 4 vs. 1 | 6.746 | 6.469 to 7.024 | Yes | **** | < 0.0001 |
|  | 5 vs. 1 | 0.428 | 0.1501 to 0.7059 | Yes | ** | 0.0066 |
|  | 6 vs. 1 | 0.7649 | 0.4870 to 1.043 | Yes | *** | 0.0003 |
|  | 3 vs. 2 | -0.8083 | -1.086 to -0.5304 | Yes | *** | 0.0002 |
|  | 4 vs. 2 | 6.412 | 6.134 to 6.690 | Yes | **** | < 0.0001 |
|  | 5 vs. 2 | 0.09375 | -0.1842 to 0.3717 | No | ns | 0.7566 |
|  | 6 vs. 2 | 0.4307 | 0.1527 to 0.7086 | Yes | ** | 0.0064 |
|  | 4 vs. 3 | 7.221 | 6.943 to 7.498 | Yes | **** | < 0.0001 |
|  | 5 vs. 3 | 0.9021 | 0.6241 to 1.180 | Yes | *** | 0.0001 |
|  | 6 vs. 3 | 1.239 | 0.9610 to 1.517 | Yes | **** | < 0.0001 |
|  | 5 vs. 4 | -6.318 | -6.596 to -6.041 | Yes | **** | < 0.0001 |
|  | 6 vs. 4 | -5.982 | -6.259 to -5.704 | Yes | **** | < 0.0001 |
|  | 6 vs. 5 | 0.3369 | 0.05898 to 0.6148 | Yes | * | 0.0212 |
|  |  |  |  |  |  |  |
| **2.4.** | **IFN-γ** |  |  |  |  |  |
|  | **ANOVA summary** |  |  |  |  |  |
|  | F | 17392 |  |  |  |  |
|  | P value | < 0.0001 |  |  |  |  |
|  | P value summary | **** |  |  |  |  |
|  | Are differences among means statistically significant? (P < 0.05) | Yes |  |  |  |  |
|  | R square | 0.9999 |  |  |  |  |
|  |  |  |  |  |  |  |
|  | **ANOVA table** | **SS** | **DF** | **MS** | **F (DFn, DFd)** | **P value** |
|  | Treatment (between columns) | 450.7 | 5 | 90.14 | F (5, 6) = 17392 | P < 0.0001 |
|  | Residual (within columns) | 0.0311 | 6 | 0.005183 |  |  |
|  | Total | 450.7 | 11 |  |  |  |
|  |  |  |  |  |  |  |
|  | **Tukey's multiple comparisons test** | **Mean Diff.** | **95% CI of diff.** | **Significant?** | **Summary** | **Adjusted P Value** |
|  | 2 vs. 1 | 0.9032 | 0.6167 to 1.190 | Yes | *** | 0.0001 |
|  | 3 vs. 1 | 0.9716 | 0.6850 to 1.258 | Yes | **** | < 0.0001 |
|  | 4 vs. 1 | 17.1 | 16.81 to 17.38 | Yes | **** | < 0.0001 |
|  | 5 vs. 1 | -0.1044 | -0.3909 to 0.1822 | No | ns | 0.7031 |
|  | 6 vs. 1 | 2.035 | 1.749 to 2.322 | Yes | **** | < 0.0001 |
|  | 3 vs. 2 | 0.06835 | -0.2182 to 0.3549 | No | ns | 0.9187 |
|  | 4 vs. 2 | 16.19 | 15.91 to 16.48 | Yes | **** | < 0.0001 |
|  | 5 vs. 2 | -1.008 | -1.294 to -0.7210 | Yes | **** | < 0.0001 |
|  | 6 vs. 2 | 1.132 | 0.8456 to 1.419 | Yes | **** | < 0.0001 |
|  | 4 vs. 3 | 16.12 | 15.84 to 16.41 | Yes | **** | < 0.0001 |
|  | 5 vs. 3 | -1.076 | -1.362 to -0.7894 | Yes | **** | < 0.0001 |
|  | 6 vs. 3 | 1.064 | 0.7773 to 1.350 | Yes | **** | < 0.0001 |
|  | 5 vs. 4 | -17.2 | -17.49 to -16.91 | Yes | **** | < 0.0001 |
|  | 6 vs. 4 | -15.06 | -15.35 to -14.77 | Yes | **** | < 0.0001 |
|  | 6 vs. 5 | 2.14 | 1.853 to 2.426 | Yes | **** | < 0.0001 |
